# Traits Explaining Durum Wheat (*Triticum turgidum* L. spp. Durum) Yield in Dry Chilean Mediterranean Environments

**DOI:** 10.3389/fpls.2017.01781

**Published:** 2017-10-20

**Authors:** Gerlitt González-Ribot, Marcela Opazo, Paola Silva, Edmundo Acevedo

**Affiliations:** Laboratory of Soil-Plant-Water Relations, Department of Agricultural Production, Faculty of Agronomical Sciences, University of Chile, Santiago, Chile

**Keywords:** morphological and physiological traits, yield components, water stress, breeding, abiotic stress

## Abstract

Yield under water stress (YS) is used as the main criterion in the selection of wheat varieties for dry Mediterranean environments. It has been proposed that selection of genotypes using YS assisted by morphological and physiological traits associated with YS is more efficient in selecting high yielding genotypes for dry environments. A study was carried out at the Antumapu Experiment Station of the University of Chile, located in Santiago, Chile (33° 40′S and 70° 38′ W). The objective was to evaluate the extent to which morpho physiological traits could explain YS. For this purpose, grain yield and yield components of 185 durum wheat genotypes from ICARDA (International Center for Agricultural Research in the Dry Areas) and INIA (Chilean National Institute for Agricultural Research) were evaluated along with seed size and weight, days to heading (DH), glaucousness (GLAU), plant height (PH) and ^13^C discrimination (Δ). The design was an α-lattice with two replications, the genotypes were grown in two different water conditions (high and low irrigation) during two seasons (2011-2012/2012-2013). Grain weight (GW) was the only yield component with high H associated with YS, but it was not associated with yield under high irrigation (YI). The combination of YI with DH+GLAU+PH+Δ+GW obtained in LI environments explained a greater fraction of YS (38%) across years; these traits had lower genotype x environment interaction than YS, they also explained a higher proportion of yield under drought than YI. None of the traits studied could replace YS in selections for grain yield. It is concluded that these traits could aid in the selection of durum wheat subject to water stress, particularly in early generations.

## Introduction

Obtaining high yielding genotypes of Durum wheat (*Triticum turgidum* L. ssp. *durum*) for different environments, especially Mediterranean rainfed areas (rainy, cold winters and dry, hot summers), is considered difficult due to high genotype x environment interaction (Annicchiarico, 2002; Acevedo and Silva, 2007). For this reason, several authors have proposed that genotype selection under Mediterranean rainfed conditions may be improved by selecting for traits associated with yield under water stress (YS) having higher heritability than yield (Donald, 1968; Acevedo, 1992; Quarrie et al., 1999; Reynolds, 2006; McIntyre et al., 2010). These traits increase transpiration, transpiration efficiency and harvest index, to increase YS (Passioura, 1977, 2006).

Yield components are agronomic traits that provide an opportunity to improve yield under water stress, along with morphological and physiological traits. The yield components which together cover all the period of crop development are the number of grains per m^2^ (GM^2^) and grain weight (GW). Most studies agree that GM^2^ best explains yield (Fischer, 1973; Mouret et al., 1991; Slafer et al., 1996, 2003, 2014; Ribaut et al., 1997), and although some studies did not find an association of GW with yield under irrigation (Craufurd et al., 1991; Slafer et al., 1996, 2014), GW was found to be associated with yield in rainfed areas (Craufurd et al., 1991; Nachit and Ketata, 1991; Ribaut et al., 1997; Araus et al., 1998; Rharrabti et al., 2001; Royo et al., 2002; Nouri-Ganbalani et al., 2009). There is a negative association between GW and GM^2^ (Slafer et al., 1996, 2014; Saeidi and Abdoli, 2015) indicating compensation, although it has been shown that in the case of wheat the compensation is not competitive (Slafer et al., 1996) and a positive association between these two yield components has also been reported (Nouri-Ganbalani et al., 2009; Motzo et al., 2010). Several studies reported GW as the yield component with the highest heritability in bread wheat and Durum wheat (Nachit et al., 1995; Royo et al., 2002; García del Moral et al., 2003; Rharrabti et al., 2003; Wang et al., 2009; Li et al., 2015). In contrast, GM^2^ has shown similar heritability to yield in bread wheat and Durum wheat (Nachit and Ketata, 1991; Acevedo, 1992) thus yield improvements under water stress using GM^2^ have had limited success, since GM^2^ is as unpredictable as yield itself (Dolferus et al., 2011).

The morphological and physiological traits reported by the literature with high heritability and used in breeding programs are, (a) early vigor, determined mostly by the size of the seed. This trait increases soil coverage and plant water use, decreasing direct evaporation of soil water (Ritchie, 1972; Acevedo and Ceccarelli, 1989; López-Castañeda et al., 1996; Richards et al., 2001, 2011; Smith et al., 2003; Reynolds et al., 2004; Reynolds, 2006; Dodig et al., 2008; Blum, 2011; Elazab et al., 2015); (b) early flowering, which provides the ability to escape to terminal drought stress (Acevedo and Ceccarelli, 1989; Quarrie et al., 1999; Richards et al., 2001; Reynolds, 2006; Reynolds et al., 2007; Araus et al., 2008; Motzo et al., 2010; Blum, 2011; Crossa et al., 2016); (c) leaf glaucousness, produced by a white wax as a result of the β-diketone that appears on the plant cuticle and reduces water loss by increasing resistance to the movement of water to the leaf surface and altering the leaf energy balance favorably due to a greater reflection of short wave radiation, decreasing leaf temperature (Quarrie et al., 1999; Richards et al., 2001; Reynolds et al., 2004, 2007; Reynolds, 2006; Saint Pierre et al., 2010; Blum, 2011; Yeats and Rose, 2013; Zhang et al., 2013; Gosney et al., 2016); (d) plant height, modern genotypes are semi-dwarfs since they carry one of the *Rht* genes (Mellado, 2007; Sip et al., 2009), and this has been positive for grain yield, however it has been found that greater plant height has a positive effect on yield in dry Mediterranean rainfed conditions; for this reason it is desirable that the genotypes maintain their height in arid rainfed areas (Zapata et al., 2004; Royo et al., 2014) compared to irrigated areas; e) ^13^C isotopic discrimination (Δ), C_3_ plants discriminate ^13^C, preferring ^12^C during CO_2_ fixation (Sarievaa et al., 2010). In wheat, Δ has a positive relation to CO_2_ intercellular concentration and a negative relation to transpiration efficiency (TE) (Acevedo et al., 1997; Araus et al., 2001, 2013; Condon et al., 2002; Rebetzke et al., 2002; Monneveux et al., 2005; Khazaei et al., 2009; Barbour et al., 2010; Sarievaa et al., 2010; Blum, 2011; Elazab et al., 2015). Although a negative association between Δ and yield has been found under very dry Mediterranean rainfed conditions, a positive association is more common because genotypes capable of sustaining greater stomatal conductance and water consumption, are more productive and better adapted (Araus et al., 2013; Elazab et al., 2015).

Yield under water stress (YS) has low heritability, and an important unexplained residual remains when classical selection criteria including yield potential, YS and flowering date are used (Bidinger et al., 1987) to select for dry environments. We need to know the proportion of YS explained by each trait, i.e., the ability of a morphological or physiological trait or combination to explain yield variation (Ceccarelli et al., 1991; Acevedo, 1992; Falconer and Mackay, 1996; Reynolds et al., 2007).

This study tests the hypothesis that Durum wheat yield growing under water stress can be improved when grain yield along with non-competitive yield components and morphological and physiological traits are used in selection. The overall objective is to determine how much YS of Durum wheat improves under Mediterranean arid conditions if grain weight along with the morpho-physiological traits seed size, flowering date, leaf glaucousness, plant height and ^13^C isotopic discrimination are present.

## Materials and methods

### Experimental design

The study was carried out in a dry environment, at the Antumapu Experiment Station of the Faculty of Agronomic Sciences of the University of Chile (33° S, 70° W, 625 m elevation). The soil is a sandy clay, alluvial Mollisol with a slope lower than 1%, good drainage and an effective depth between 30 and 73 cm. 185 unrelated high-yield Durum wheat genotypes (Annex I in Supplementary Material) of different origins (ICARDA and INIA) adapted to Mediterranean conditions were sown in a 14 × 14 α-lattice design with two replications for two seasons (2011-2012/2012-2013), under 2 different water conditions (high irrigation and low irrigation). The experimental unit (EU) was a plot of 2 m^2^ (1 m wide × 2 m long) having five 2 m long and five 20 cm apart rows.

### Agronomic management

Planting was done on May 31 of each year; about 382 seeds per m^2^ were sown manually at a depth of 3 cm. Weeds were controlled manually during the two seasons; there were no crop pests or diseases to be controlled in either season. Fertilization each season followed soil analysis and yield expectations for the site (yield target 5,000 kg/ha and 12% protein). To determine the timing and amount of irrigation, soil water content was evaluated twice per week with FDR probes (Frequency Domain Reflectometry Diviner 2000 probe), inserting two 60 cm depth access tubes in 2 plots per replication of each water condition. Climatic data were obtained from the nearby (500 m) weather station at La Platina, INIA (33° S, 70° W, and 625 m elevation) next to the test area.

Accumulated rainfall for the first season was 173 mm, which occurred between June and September; there was no rain in the sowing month (May), thus 15 mm of water were applied to facilitate sowing. In September rainfall was very low (5 mm), so irrigation (sprinkler) started at that time. In the second season rainfall was 230 mm, accumulated between May and November; the months of July, September and November had very little rain, thus irrigation began in August. The low irrigation trial was partially irrigated to avoid crop failure. The total water received by the low irrigation and high irrigation plots in the first year was 263 and 413 mm, and in the second year 275 and 440 mm, respectively (Table 1).

**Table 1:** Rainfall and Water applied (mm) monthly for the two irrigation treatments (environments).

**Month**	**Year 1**			**Year 2**		
	**Rain**	**LI**	**HI**	**Rain**	**LI**	**HI**
May		15.0	15.0	51.8		
June	74.0			87.9		
July	49.0			6.1		
August	45.0			40.3	15.0	15.0
September	5.0	15.0	45.0	1.8	30.0	60.0
October		60.0	90.0	42.0		45.0
November			90.0	0.1		90.0
**Total**	**173.0**	**90.0**	**240.0**	**230.0**	**45.0**	**210.0**

*Low irrigation (LI) and high irrigation (HI). The low irrigation plots simulate rainfed conditions*.

### Evaluations

Yield, its components and morphological and physiological traits were evaluated in each genotype and each environment using the following criteria.

#### Yield and its components

Grain yield (kg ha^−1^) was determined by the weight of grains obtained from mechanical threshing spikes harvested manually in 3 rows of 1.6 m length (0.96 m^2^) in the central area of each experimental unit (EU) to avoid edge effect. The yield components determined were grain weight (GW), biomass (BIO, above ground biomass), harvest index (HI), grains per spike (GS), spikes per m^2^ (SM^2^), grains per m^2^ (GM^2^), grain production rate (GPR, grain yield divided by the number of days from heading to physiological maturity) and biomass production rate (BPR, total above ground biomass divided by the number of days from sowing to physiological maturity). It was assumed that the small area harvested would increase grain yield similarly in all genotypes.

#### Morphological and physiological traits

*Seed size* was evaluated by measuring the weight (SW) and length of 10 seeds of each genotype (López-Castañeda et al., 1996). Grain weight (GW) was measured at harvest.

##### Flowering date

Anthesis in Durum wheat is difficult to recognize, since the anthers of some genotypes are not visible; for this reason we measured the number of days from sowing to heading, determining this state when the 75% of the EU had the spikes completely emerged (Zadoks 59) (Zadoks et al., 1974). The number of days between sowing and heading corresponds to days to heading (DH).

##### Physiological maturity

The date of physiological maturity was determined when 75% of the EU showed a yellow color of the flag leaf, the grains had a dry appearance and pinching them did not leave a mark (Zadoks 92), the number of days between sowing date and physiological maturity date (DM) corresponds to days to physiological maturity (Zadoks et al., 1974).

*Glaucousness* (GLAU) was recorded visually on a 1 to 5 scale (notes 1 and 5 for the least and most glaucous respectively) at Zadoks 59 (Zadoks et al., 1974), when it was at its maximum expression in leaves, stems, and spikes (Richards et al., 2001).

*Plant height* (PH) was measured in 5 plants of each EU at the end of the growing period from the ground to the apex of the main stem, without considering the awns (Zapata et al., 2004; Castañeda-Saucedo et al., 2009).

##### ^*13*^C discrimination

Carbon discrimination analysis was performed on samples of 52 randomly selected genotypes; 10 g of grain at harvest were separated and dried in a stove at 70°C for 48 h and then encapsulated in tin capsules for ^13^C composition isotope analysis. The samples were sent to the Stable Isotope Facility of the University of California at Davis, U.S.A. to be analyzed using a PDZ Europe ANCA-GSL elemental analyzer in interface with an isotope ratio mass spectrometer (IRMS) PDZ Europe 20-20 (Sercon Ltd., Cheshire, UK). Values of δ^13^C (product) obtained were used to calculate ^13^C isotopic discrimination (Δ) as follows:

(1) $$\begin{array}{r} \Delta =\frac{\delta^{13}C\;\left(source\right)-\delta^{13}C\;\left(product\right)}{1+\delta^{13}C\;\left(source\right)÷1000} \end{array}$$

Atmospheric δ^13^C is usually between 6.5 and 8.0‰, according to geographical location and altitude (Acevedo et al., 1997; Monneveux et al., 2006). A 7‰ was used in this case.

The proportion of grain yield explained by a given trait in the presence of all possible combinations of the other traits was calculated using the following formula (Ceccarelli et al., 1991; Acevedo, 1992; Reynolds et al., 2007).

(2) $$\begin{array}{r} \%\;Yield\;explained\;by\;trait=\\ \;\frac{\left[Yield\;with\;trait\;\left(+\right)-Yield\;with\;trait\;\left(-\right)\right]}{\left(10\%\;Higher\;yield-10\%\;Lower\;yield\right)} \end{array}$$

10% Higher yield refers to the highest 10% yield of the nursery and 10% Lower yield to the lowest 10% yield. Yield with trait (+) is the mean yield of genotypes having a positive expression of a trait or combination of traits. Yield with trait (−) is the mean yield of the nursery having a negative expression of a trait or combination of traits.

The data used in the calculation of the proportion of grain yield explained by a given trait were obtained through selections made by a selection index software of CIMMYT (1999) for the different combinations of the traits. To make the selection it is necessary to specify the target and intensity for each trait included in the combination, where the target is the objective which is pursued in the selection, expressed in units of standard deviation of the mean (from −3.0 to 3.0); the intensity reflects the relative importance of the different variables to be used in selection expressed in values from 0 to 10; the trait that you want to progress more must receive higher intensity (CIMMYT, 1999).

#### Statistical analysis

For statistical analysis we used the InfoStat statistical software (Di Rienzo et al., 2008), which has a friendly interface of the R platform to estimate the General Linear Mixed Model, which allows differentiating random effects from fixed effects (Di Rienzo et al., 2011). Genotypes, years, water conditions and the interaction between them were introduced in the model as fixed or random effects depending on the objective of the analysis. When the objective was the comparison of the tested material for selection, the fixed model was used. To control the experimental error from random effects the cross effects of row and column of the α-lattice design were introduced in the model as random effects nested within the blocks, which in turn were nested within the year-water condition effect. These models best fit the characteristics of this experiment. The presence or absence of significant variance was determined (*p* ≤ 0.05) in terms of traits, genotypes, environments, and interactions. For ^13^C isotope discrimination, we used 52 out of the 185 genotypes what allowed the original lattice design to be maintained. Therefore the analysis of variance for ^13^C isotope discrimination was carried out similarly to the other traits but with a lower number of genotypes (*n* = 52). The percentage of the total of the sum of squares corresponding to each source of variation in each variable was calculated. The variances needed to calculate broad sense heritability (H) of each trait were calculated using the random model and the following relation:

(3) $$\begin{array}{r} H=\frac{\sigma_{g}^{2}}{\sigma_{g}^{2}+\frac{\sigma_{ge}^{2}}{e}+\frac{\sigma^{2}}{re}} \end{array}$$

where r = number of replications, e = number of environments, σ^2^ = error variance, $\sigma_{g}^{2}=$ genetic variance, $\sigma_{ge}^{2}$ = variance of genotype X environment interaction (Falconer and Mackay, 1996).

Pearson correlations were calculated using the statistical software InfoStat (Di Rienzo et al., 2008) to determine the existence and magnitude of associations (*p* ≤ 0.05) between yield and traits; the software was also used to perform multiple linear regressions to estimate yield, using the traits and hence to determine how much the residuals changed with the presence or absence of a trait.

The worth of a trait for plant improvement was tested with the following relation (Falconer and Mackay, 1996).

(4) $$\begin{array}{r} Cry/Dry=r_{\text{G}}sqrH_{\text{c}}/H_{\text{s}} \end{array}$$

Where r_G_ is the genotypic correlation between the indirect trait (Cry) and the direct trait (Dry), H_c_ represents H of the indirect trait and H_s_ represents H of the direct trait.

## Results

### Associations between traits and yield

Table 2 shows the Pearson correlations with grain yield of the variables of concern for all environments. All variables were associated with yield in at least one year under the low irrigation treatment, but not all the variables were associated with yield under high irrigation. In all environments yield components had higher association with yield than the morphological and physiological traits, except for grain weight, which although associated with yield in both years under low irrigation, was not associated with yield in either year when the crop was fully irrigated. Δ was the only physiological trait associated with yield under high irrigation.

**Table 2:** Mean of yield, its components and morphological and physiological traits in each environment, Pearson correlation (r) of the morpho-physiological traits with yield in each environment; percent of genotypic variability (G); genotype x environment interaction (GxE) of the total sum of squares of the analysis of variance for each variable calculated with the fixed linear mixed model.

**Variable**	**LI Year 1 (263 mm)**		**LI Year 2 (275 mm)**		**HI Year 1 (413 mm)**		**HI Year 2 (440 mm)**		**G (%)**	**GxE (%)**
	**Mean**	***r***	**Mean**	***r***	**Mean**	***r***		***r***		
GY	2,502		2,899		6,427		6,475		24^***^	34^*^^a^^b^
GW	37.3	0.33^***^	33.9	0.47^***^	54.4	ns	57.6	ns	48^***^	17^*^^a^
BIO	10,005	0.60^***^	14,228	0.65^***^	20,214	0.77^***^	18,793	0.65^***^	22^*^	23^*^^a^
HI	0.25	0.86^***^	0.20	0.82^***^	0.32	0.82^***^	0.35	0.63^***^	31^***^	39^*^^a^^b^
SM^2^	414	0.28^**^	512	0.24^**^	526	0.46^***^	530	0.45^***^	37^***^	44^*^^a^^c^
GM^2^	6,751	0.88^***^	8,648	0.71^***^	11,986	0.90^***^	11,340	0.87^***^	43^***^	23^*^^a^
GS	16.4	0.83^***^	17.1	0.54^***^	23.0	0.65^***^	21.8	0.47^***^	39^***^	41^*^^a^^b^
GPR	67.5	0.97^***^	71.1	0.92^***^	145.6	0.94^***^	119.8	0.93^***^	23^***^	33^*^^a^^b^
BPR	63.9	0.60^***^	91.0	0.68^***^	125.0	0.76^***^	111.5	0.68^***^	22^*^	21^*^^a^
SW	58.3	ns	54.4	0.22^***^	58.3	ns	54.4	ns	68^***^	32^*^^a^
DH	137	−0.38^***^	129	−0.29^***^	134	ns	126	ns	58^***^	17^*^^a^
GLAU	3.5	0.22^**^	3.7	ns	3.6	ns	3.6	ns	56^***^	24^*^^a^
PH	74.1	0.34^***^	98.6	ns	103.9	ns	101.1	ns	10^***^	ns
Δ	16.1	0.46^***^	17.9	0.26ns	18.3	0.55^***^	18.6	0.30^*^	27^**^	ns
YI		ns		0.31^***^		0.21^***^	6,475	1.00		
YS	2,502	1.00		ns		0.20^**^		ns		

*GY, grain yield (kg/ha); GW, weight of grains (g); BIO, biomass (kg/ha); HI, harvest index; SM^2^, spikes per m^2^; GM^2^, grains per m^2^; GPR, grain production rate (kg/ha/d); BPR, biomass production rate (kg/ha/d); SW, seed weight (g); DH, days from sowing to heading; GLAU, glaucousness (visual scale); PH, plant height (cm); Δ, ^13^C isotopic discrimination (‰); YI, maximum yield under irrigation (irrigation year 2); YS, yield in more stressed environment (rainfed year 1)*.

*** p ≤ 0.001;

** p ≤ 0.01;

* p ≤ 0.05; ns, not significant (n = 185, except Δ with n = 52). GxE, percent of the total of the sum of squares of the analysis of variance corresponding to significant interactions;

a , data correspond to genotype-year interaction;

b , genotype-water condition interaction;

c *, genotype-year-water condition interaction; two letters together, data correspond to the sum of the percentages of the significant interactions*.

We analyzed the correlation between years of yield and the variables under low irrigation. The results are shown in Table 3, where it can be seen that traits were correlated between years under low irrigation except for yield (*p* ≤ 0.10).

**Table 3:** Pearson correlation coefficients among years of yield and of high heritability variables under low irrigation.

**Variable**	**Pearson correlation coefficient**
GW	0.20^**^
SW	0.33^***^
DH	0.47^***^
GLAU	0.26^***^
PH	0.30^***^
Δ	0.22^†^
YI	0.21^**^
YS	NS

*GW, weight of grains (g); SW, seed weight (g); DH, days from sowing to heading; GLAU, glaucousness (visual scale); PH, plant height (cm); Δ, ^13^C isotopic discrimination (‰); YI, maximum yield under irrigation (irrigation year 2); YS, yield in more stressed environment (rainfed year 1)*.

*** p ≤ 0.001;

** p ≤ 0.01;

^*^p ≤ 0.05;

† *p ≤ 0.10; NS, not significant (n = 185 except Δ with n = 52)*.

Seed length was measured in the first year only and was correlated (*r* = 0.60) with seed weight (SW) in that year, while SW was correlated with grain weight (GW) in both environments with a Pearson correlation coefficient above 0.33; in addition, a negative association was found between GM^2^ and GW in all environments (Table 4).

**Table 4:** Pearson correlation coefficients among grain variables in each environment.

**Variables**	**LI**		**HI**	
	**Year 1**	**Year 2**	**Year 1**	**Year 2**
GW and GM^2^	−0.15^*^	−0.28^*^	−0.47^*^	−0.46^*^
GW and SW	0.37^*^	0.33^*^	0.38^*^	0.38^*^
SW and LS	0.60^*^	nd	nd	nd

*GW, grain weight; GM^2^, grains per m^2^; SW, seed weight; LS, length of seed*.

* *p ≤ 0.05; nd, no data (n = 185)*.

### Trait contributions to yield under water stress

To assess how much each trait contributed to yield under low irrigation, multiple linear regressions of the traits including YI, with yield under low irrigation as the dependent variable were performed for each year. The residuals of the multiple regressions corresponded to the yield variation not explained by the regression; the best regression been the one with the smallest residual. The analysis was performed with the 52 genotypes in which Δ was measured, considering the traits listed in Table 5.

**Table 5:** Percentages of the residuals of the sum of squares of the multiple linear regression along with the coefficient of determination.

**Independent variables**	**LI year 1**		**LI year 2**		**Residuals difference (%)**
	**Residual (%)**	***R*^2^**	**Residual (%)**	***R*^2^**	
DH+YI	98	0.02^NS^	81	0.19^**^	17
DH+YI+GW	93	0.07^NS^	81	0.19^*^	12
DH+YI+SW	85	0.15^*^	68	0.32^***^	17
DH+YI+GLAU	95	0.05^NS^	76	0.24^**^	19
DH+YI+PH	92	0.08^NS^	79	0.21^**^	13
DH+YI + Δ	76	0.24^**^	77	0.23^**^	1
DH+YI+GLAU+PH + Δ	64	0.36^***^	74	0.26^*^	10
DH+YI+GLAU+PH + Δ + SW	54	0.46^***^	71	0.29^*^	17
DH+YI+GLAU+PH + Δ + GW	62	0.38^***^	62	0.38^***^	0
DH+YI+GLAU+PH + Δ + GW + SW	51	0.49^***^	62	0.38^**^	11

*DH days to heading; YI, maximum yield with HI irrigation; GW, grain weight; SW, seed weight; GLAU, glaucousness; PH, plant height; Δ, ^13^C discrimination. Difference in the residuals between the two years*.

*** p ≤ 0.001;

** p ≤ 0.01;

* *p ≤ 0.05; NS, not significant (n = 52)*.

*The dependent variable each year was grain yield under low irrigation (LI)*.

Using classical selection criteria (YS = DH+YI) the minimum residual was 81%, significant only for one of the 2 years under low irrigation. The residuals were reduced adding traits independently, but the reduction was significant only in one of the years, except for SW and Δ, whose reduction was significant in both years under low irrigation. The combination of two or more traits reduced the residuals to less than 74%, reaching 51% in the first year when all traits were combined (Table 5). The traits along with DH and YI that most decreased the residuals were GLAU, PH, Δ and GW, with no differences between years (Table 5).

### Percentage of YS explained by each trait or combination

Table 6 shows the proportion of YS explained by each trait separately in the year of greater water stress, the target of selection was + 3 and −3, and the intensity was 10 for each of these traits. The traits with the lowest explanation of YS were SW and YI (0.06 and 0.01, respectively), and those with the higher explanation of YS were Δ and GW, followed by PH and GLAU.

**Table 6:** Explanation of YS by trait selecting 10% of the total genotypes having maximum and minimum expression of each character, and the 10% of the genotypes of maximum and minimum yield in the year of greater water stress (Equation 2).

**Trait**	**Explanation of YS**
GW	0.37
SW	0.06
DH	0.34
GLAU	0.23
PH	0.29
Δ	0.37
YI	0.01

*GW, grain weight; SW, seed weight; Δ, DH, days to heading; GLAU, glaucousness; PH, plant height; ^13^C discrimination; YI, maximum yield high irrigation. n = 185 except Δ with n = 52*.

Table 7 shows the proportion of YS explained by combinations of the traits in the year with greater water stress. All traits were considered, including Δ, so the corresponding population was 52 genotypes, target was + 3 and −3, and intensity 10. Almost all the combinations shown in this table explain a higher proportion of YS than any trait individually (0.37 in Table 6); except for those in which YI is added to the combination (0.35) and Δ combined with DH (0.31). Of the 3 greatest explanations of YS, the combination Δ+DH+GLAU+PH has the greatest number of traits.

**Table 7:** Explanation of YS by combinations of traits selecting 10% of 52 genotypes having maximum and minimum expression of each combinations obtained under LI environments, and the 10% of the genotypes of maximum and minimum yield in the year of greater water stress.

**Combinations**	**Explanation of YS**
Δ + DH	0.31
Δ + GLAU	0.59
Δ + PH	0.48
Δ + DH + GLAU	0.56
Δ + DH + GLAU + PH	0.49
Δ + DH + GLAU + PH + GW	0.40
Δ + DH + GLAU + PH + GW + YI	0.35
Δ + GLAU+PH	0.44
Δ + GLAU+PH+GW	0.41
Δ + PH + GLAU + GW + YI	0.24

*Δ, ^13^C discrimination; DH, days to heading; GLAU, glaucousness; PH, plant height; GW, grain weight; YI, maximum yield high irrigation*.

The genotypes of Durum wheat showing the highest expression of each of the traits of the combination Δ+DH+GLAU+PH were in the environment of greater water stress. Note that lower values of DH are considered greater expression. These genotypes showed yields in the Mediterranean low irrigation conditions above the mean (2,502 kg/ha) of the original population (185 genotypes). The number of each genotype is given in Table 8 and its description in the Annex I; these genotypes are ordered within columns from greater to lower trait expression. Genotypes marked with ^*^ are among the 5 genotypes of greatest YS out of a total of 185.

**Table 8:** Durum wheat genotypes selected by the combination of the maximum expression of the traits ^13^C discrimination, glaucousness, plant height and days to heading, in the environment of greater water stress (*n* = 185, except for the genotypes with ^13^C discrimination determination, *n* = 52).

^**13**^**C discrimination**		**Days to heading**		**Glaucousness**		**Plant height**		**Combination**	
**Genotype**	**Yield (kg/ha)**	**Genotype**	**Yield (kg/ha)**	**Genotype**	**Yield (kg/ha)**	**Genotype**	**Yield (kg/ha)**	**Genotype**	**Yield (kg/ha)**
53^*^	3,916	89	2,756	171	2,579	98	3,264	98	3,264
2	2,826	62	3,594	111	3,369	139	3,505	97	3,271
77	2,600	173	2,943	137^*^	4,698	48	3,290	111	3,369
20	2,942			63	3,142	40^*^	3,642	40^*^	3,642

* *, Among the 5 highest yielding genotypes (n = 185). Column order from highest to lowest trait expression. The genotypes are described in the Annex I*.

### Implications for plant breeding

Traits useful for breeding plants for water stressed environments are required to have a high genetic correlation with YS along with high heritability. Formula 4 from Falconer and Mackay (1996) puts these two concepts together. Table 9 shows the ratio of indirect (selection for a trait other than grain yield) vs direct selection (selection for yield under water stress) and shows that selection for SW, DH, GLAU, PH and Δ, all make a positive contribution to selection for grain YS (recall that lower days to heading is the desirable trait for DH in Mediterranean environments). The positive contribution of these traits occurs under water stress only. The last column of Table 9 shows that the contribution drops to practically zero or is even negative under high irrigation. The heritabilities in this table were calculated with the random linear mixed model.

**Table 9:** Genetic correlation (r_g_) of each trait with yield in low irrigation environments.

**Variable**	**rg for each trait with GY Low irrigation (263–275 mm)**	***G* (%)**	**GxE (%)**	***H***	**CRx/CRy YS/Direct Trait**	**CRx/CRy YI/Direct trait**
GY(stress)	–	45.6^**^	43.9^**^	0.24		
GW	0.28^***^	48.4^***^	37.4^**^	0.51	0.41	−0.10
BIO	0.63^***^	11.2^ns^	16.5^*^	0.09	0.39	0.33
HI	0.87^***^	38.6^+^	35.6^ns^	0.23	0.85	0.87
SM^2^	0.17^**^	31.9^*^	30.1^***^	0.26	0.18	0.32
GM^2^	0.80^***^	42.8^***^	28.9^ns^	0.41	1.05	0.91
GS	0.78^***^	60.9^***^	37.7^ns^	0.34	0.93	0.70
GPR	0.94^***^	49.3^**^	49.3^**^	0.21	0.88	0.82
BPR	0.64^***^	11.5^ns^	15.6^+^	0.11	0.43	0.39
SW	0.15^*^	55.6^***^	28.3^***^	0.70	0.26	0.00
DH	−0.41^***^	24.4^***^	9^ns^	0.61	−0.65	−0.12
GLAU	0.13^*^	59.2^***^	38.4^*^	0.56	0.20	−0.05
PH	0.25^***^	11.4^***^	6.4^ns^	0.43	0.33	−0.02
Δ	0.19^**^	45.7^ns^	25.1^ns^	0.19	0.17	0.06

*Broad sense heritability (H), percentage of the total sum of square for G and GxE for each variable and merit of indirect selection relative to that of direct selection (CRx/DRy). Low irrigation was taken as the mean of each trait in the two years of the experiment*.

*YI is the grain yield under full irrigation, YS is the grain yield under stress, GxE is the genotype x environment interaction, GY, grain yield (kg/ha); GW, weight of grains (g); BIO, biomass (kg/ha); HI, harvest index; SM^2^, spikes per m^2^; GM^2^, grains per m^2^; GS, grains per spike; GPR, grain production rate (kg/ha/d); BPR, biomass production rate (kg/ha/d); SW, seed weight (g); DH, days from sowing to heading; GLAU, glaucousness (visual scale); PH, plant height (cm); Δ, ^13^C isotopic discrimination (‰); YS, yield in more stressed environment (rainfed year 1)*.

*** p ≤ 0.001;

** p ≤ 0.01;

* p ≤ 0.05;

+ *p ≤ 0.10; ns, not significant (n = 185, except Δ with n = 52). GxE, percent of the total of the sum of squares of the analysis of variance corresponding to significant interactions*.

None of the indirect traits by itself will lead to higher yield (Table 9) and they may be only used in early stages of selection and only when trials are conducted under water stress conditions.

## Discussion

A considerable decrease in durum wheat yield occurs with lower than 300 mm annual rainfall which is associated with greater genotype x environment interaction (Annicchiarico, 2002). When breeding for high yielding genotypes in these environments, the use of indirect selection traits, associated with greater grain yield under water stress, having lower genotype × environment interaction would make results more reliable and repeatable in most of the environments (Falconer and Mackay, 1996).

The criteria for choosing a trait of indirect selection included genetic variability, easy to measure, association with the direct trait (grain yield), high heritability and low genotype x environment interaction (Falconer and Mackay, 1996; Reynolds et al., 2007). Traits which met these criteria were grain weight, days to heading, glaucousness, plant height and Δ (Tables 2, 9).

All agronomic traits, except SM^2^ had high merit for indirect selection under the dry environment (Table 9). GW is not competitive with other traits, making wheat improvement using other agronomic traits relatively useless. The highest heritabilities (Table 9) were obtained for the morpho physiological traits, except for Δ which had a very low H (0.19).

Although the number of grains (GM^2^) has been traditionally used to improve wheat yield under irrigation (Slafer et al., 1996, 2014), and shows a high correlated response with grain yield under stress (Crx/Cry, YS Table 9), it is extremely vulnerable to stress, which causes abortion and floret sterility, affecting the number of grains (Dolferus et al., 2011). Because of this, yield improvement by selection using GM^2^ has had limited success in stress environments (Dolferus et al., 2011).

Another important point to consider in the choice of traits to combine guiding the selection process is their association with YS (Table 9). All yield components had a significant genotypic correlation with yield under low irrigation but they lose their value due to the compensatory interactions with other yield components. There was no association of GW with yield under high irrigation (data not shown), in accordance with other studies (Slafer et al., 1996, 2014), instead grain weight was associated with yield under low irrigation (Tables 2, 9) which has also been reported in other studies (Reynolds, 2006). Days to heading was negatively correlated with yield, indicating that the most precocious genotypes would be desirable, in accordance with other reports for Mediterranean environments (Acevedo and Ceccarelli, 1989; Quarrie et al., 1999; Richards et al., 2001; Reynolds, 2006).

According to Table 2 some traits were associated with yield under low irrigation in one year but not in the other, what could result from the inter-annual variation of yield and not of the traits. Table 3 shows that yield under low irrigation was different between years; by contrast all indirect traits correlated with LI yield in one year were also correlated between years under LI (Table 3), demonstrating that this effect was mainly due to the inter-annual variation of YS and no to the variation of the traits. Furthermore, the merit of the indirect selection traits holds under water stress only (last two columns, Table 9).

As reported by other authors (López-Castañeda et al., 1996; Smith et al., 2003) we found that seed weight was associated with seed length (Table 4). The correlation found between seed weight (SW) and grain weight (GW) indicates that sowing large seeds will result in large grains at harvest (Richards et al., 2001; Reynolds et al., 2004; Reynolds, 2006; Sadras, 2007; Table 4). If genotypes of greater GW are chosen in the first generation, in the second generation there will be greater GW under water stress, since GW has high heritability (Table 9). GW would be a good estimator of grain weight at sowing (SW) and YS (Table 2).

There was a negative relation between GW and GM^2^ in all environments of this study (Table 4), which indicates compensation between GW and GM^2^, thus trying to improve YS using GM^2^ would be counterproductive because individual grain weight will be reduced. It has been argued that this compensation is not competitive (Slafer et al., 1996), i.e., there is no competition between grains for available assimilates, since these components are determined at different growth stages of the crop; GM^2^ is determined before GW begins and there is no feedback, thus theoretically variation in GM^2^ should have a minor effect on GW and grain size could be increased while maintaining the number of grains under stress conditions (Motzo et al., 2010). This was not the case in this study.

Indirect traits selected that complied with the criteria to be used in plant breeding for YS included days to heading (DH), glaucousness (GLAU), plant height (PH), ^13^C Discrimination (Δ), grain weight (GW) and seed weight (SW).

In plant breeding programs for dry Mediterranean environments the trait most used is flowering date in conjunction with yield potential, but while it is true that flowering date has high merit for selection and is negatively associated with yield in Mediterranean environments (DH, Table 9) when improving using these criteria much unexplained residual remained, thus it was important to know what would happen with the residual if one or all traits were added to a multiple linear regression (Table 5). The indirect selection traits for YS chosen in this study, GLAU, PH, Δ, GW, and SW, together with DH determined under drought stress and yield under full irrigation (YI) decreased the residuals (Table 5). When these traits were combined, the greatest decrease in residuals was the one that contained all the traits except seed weight (SW) (Table 5). When GW was not introduced in the combination, the residuals increased, as well as the residuals difference between years, showing the importance of grain weight in the combination. Thus the traits ^13^C discrimination (Δ), plant height (PH), glaucousness (GLAU), and grain weight (GW) combined with yield under irrigation (YI) and flowering date (DH) were those which explained a greater fraction of yield under low irrigation (Table 5).

The measure of the percentage of the overall yield explained by a trait is an index that gives a measure of how good the trait is to explain yield indirectly (Ceccarelli et al., 1991). The objective of using traits to select yield under stress indirectly is to achieve an increase in yield heritability and a decrease in the genotype x environment interaction, thus understanding what trait or combination of traits explains a higher percentage of YS allows choosing those which most contribute to yield and will lead to better selection with greater contribution to heritability (Table 9).

By using selection criteria for each single trait we achieved explanation of YS greater than 0.23 in almost all the traits except for seed weight (0.06) and yield under HI (0.01) (Table 6). When YI was included in trait combinations the percent of explanation of YS decreased (Table 7) despite being correlated with yield under low irrigation, indicating that it is not a good indirect selection trait for genotypes under water stress. While the morpho-physiological traits together without considering YI (Table 7) always showed a higher explanation of YS than separately (Table 6), the use of more traits did not significantly improve the explanation of YS (Table 7). This is not to say that more traits should not be used, because the contribution of the traits in terms of heritability (Table 9) should also be considered. The greatest explanation of YS (0.59) was obtained by combining Δ and glaucousness (GLAU), and it was followed by the combination that also contained DH (0.56) (Table 7). Although these appear to be the best combinations, analyzing trait heritabilities (Table 9), it is clear that the gain in explanation of YS does not imply better heritability, therefore it would be preferable to use the combination that does not significantly decrease explanation of YS and employs the largest number of traits with high heritability, as in the case of the combination Δ + DH + GLAU + PH (Table 7). While it is true that using a large number of traits increases economic cost, to obtain the best estimate one must choose between the more effective method (combined selection) and the less expensive method (single selection) (Falconer and Mackay, 1996).

In crop improvement by backcrossing the final product is a line containing the gene of interest from the donor parent, with the genotype of the recurrent parent in the rest of the genome (Hospital, 2005). Therefore, a good donor genotype for a trait is one that provides its best expression, because the recurrent parent provides the rest of the genome. This study allowed us to identify genotypes of Durum wheat as donors of each trait of the best proposed combination (Δ+DH+GLAU+PH) for higher YS (Table 8). Some of these genotypes are also the genotypes with greatest yield in the low irrigation conditions (genotypes 40, 53, and 137). To understand in detail the features of each genotype selected, the Annex I provides a description of each of the 185 genotypes used in this study.

## Conclusions

The selection for yield under drought of Durum wheat under Mediterranean arid conditions, using grain weight along with the morpho-physiological traits seed size, flowering date, leaf glaucousness, plant height and ^13^C isotopic discrimination can assist in selection for grain yield particularly in early generations but the nursery must be grown under drought stress. Possible donor parents of each trait of the best proposed combination (Δ+DH+GLAU+PH) for higher YS were identified.

None of the indirect selection traits tested here will lead per *se* to higher grain yield. They may assist in the selection process, particularly in early generations.

The hypothesis that selection of Durum wheat YS improves when grain yield along with non-competitive yield components and morphological and physiological traits are used is sustained.

## Author contributions

GG and EA conceived the experiment, GG, PS, and MO conducted the Experiment, GG analyzed the results. GG and EA wrote the manuscript. All authors reviewed the manuscript.

### Conflict of interest statement

The authors declare that the research was conducted in the absence of any commercial or financial relationships that could be construed as a potential conflict of interest.
