# Supplementary material for: Traits Explaining Durum Wheat (Triticum turgidum L. spp. Durum) Yield in Dry Chilean Mediterranean Environments
Source: Front Plant Sci. 2017 Oct 20;8:1781. doi: 10.3389/fpls.2017.01781 (PMC5654942; doi:10.3389/fpls.2017.01781)
Supplement: Supplementary file 1 [file DataSheet1.pdf]

Annex I

Description of the Durum wheat genotypes used in the study.

GENO-TYPE	CODE	CROSSES	PEDIGREE	ORIGIN
1	LLARETA	D67.54.4.9A//JORI'S'/ROSNER DURUM 119-200-4Y/3/ SAHEL77	CD-64399-4P-2P-3P	INIA
2	CORCOLEN	ALGA'S'/3/CANDEALFEN5/FLAMINGO'S'//PETREL'S'/4/CHURRILLAS'S'/5/AUK'S'/6/RUFF'S'/FLAMINGO'S'//FLAMINGO'S'/CRANE'S'/3/YAV79/HUITLES'S'	A-27357-0P-5P-3P-3P	INIA
3	LLEUQUE	YEL'S'/BAR'S'/3/GR'S'/AFN//CR'S'/5/DOM'S'//CR'S'*2/GS'S'/3/SCO'S'/4/HORA/6/LAP76/GULL'S'/7/KICAN	A-27735-0P-3P-1P-1P	INIA
4	QUC 3104-2005	ALTAR84/ALD'S'//STN'S'/CHEN'S'/ALTAR84/4/ATES1D	A-30064-0P-0P-0P-2P	INIA
5	QUC 3497-2007	NACH'S'/CHEN'S'//RUFO'S'/ALD'S'/3/SQLA'S'/7/YEL'S'/BAR'S'/3/GR'S'/AFN//CR'S'/5/DOM'S'//CR'S'*2/GS'S'/3/SCO'S'/4/HORA/6/ LAP76/GUIL'S'	A-31604-0P-0P-0P-0P-5A-0C	INIA
7	QUC 3672-2008	SNITAN/3/STOT//ALTAR84/ALD	CDSS99Y00645S-0M-0Y-111Y-0M-0Y-1M-0Y	INIA
8	QUC 3693-2008	GUAYACAN INTA//YUAN1/GREEN18/3/SOOTY 9/RASCON 37	CDSS00Y01041T-0TOPB-8Y-0BLR-5Y-0B-0Y-2M-0Y	INIA
9	QUC 3694-2008	GUANAY/3/STOT//ALTAR84/ALD/4/BINTEPE85/SULA	CDSS00Y01051T-0TOPB-14Y-OBLR-1Y-0B-0Y-1B-0Y	INIA
10	QUC 3738-2008	OSU-3880005/3/STOT//ALTAR84/ALD/4/KUCUK2/5/RASCON 37/2*TARRO2	CDSS02Y00536S-0Y-0M-20Y-0Y	INIA
11	QUC 3739-2008	OSU-3880005/3/STOT//ALTAR84/ALD/4/KUCUK2/5/CRAKE10/RISSA	CDSS02Y00540S-0Y-0M-22Y-0Y	INIA
12	QUC 3755-2008	VANRRIKSE6.2//1ª-1D 2+12-5/3*WB881	CDSS98Y00665S-0M-5Y-0M-0Y-0BLR-1Y-0B	INIA
13	QUC 3757-2008	STOT//ALTAR84/ALD/3/GREEN18/FOCHA1//AIRON1	CDSS99B00467S-0M-0Y-75Y-0M-0Y-2M-0Y	INIA
14	QUC 3775-2008	ATES 1-D/LLARETA INIA	A-31865-0P-0P-0P-1A-1C-0C	INIA
15	QUC 3347-2006	YEL'S'/BAR'S'/3/GR'S'/AFN//CR'S'/5/DOM'S'//CR'S'*2/GS'S'/3/SCO'S'/4/HORA/6/LAP76/GUIL'S'/7/ALTAR84/CMH82A.1062//RISSA'S'	A-31213-0P-0P-0P-0P-4P-0C	INIA
16	QUC 3698-2007	AINZEN1//HYDRANASSA30/SILVER5/3/AUK/GUIL//GREEN	CDSS00B00306T-0TOPY-0B-15Y-0M-0Y-1B	INIA

17	QUC 3730-2008	AJAIA3/ANADE1//AFUWAN14/7/ECO/CMH76A.722//YAV/3/ALTAR84/4/AJAIA2/5/KJOVE1/6/MALMUK1/SERRATOR1	CDSS02Y00280S-0Y-0M-7Y-0Y	INIA
18	QUC 3731-2008	KUCUK2/PATA2/4/ARMENT//SRN3/NIGRIS4/3/CANELO9.1	CDSS02Y00306S-0Y-0M-26Y-0Y	INIA
19	QUC 3763-2008	LLARETA INIA/YEBAS8/3/MINIMUS6/PLATA16//IMMER	CDSS00Y01047T-0TOPB-5Y-0BLR-1Y-0B-0Y-1B-0Y	INIA
20	QUC 3767-2008	LLARETA INIA/ATES 1-D	A-32128-0P-0P-1A-1C-0C	INIA
21	QUC 3769-2008	LLARETA INIA/3/CD64399-4P-2P-3P//STN’S’/GOTE’S’	A-32139-0P-0P-1A-1C-0C	INIA
22	QUC 3782-2008	POHO1/4/ALTAR84/CMH82A.1062//RISSA’S’/3/ACONCHI 89	A-31900-0P-0P-0P-1A-3C-0C	INIA
23	QUC 3788-2008	GUAYACAN-INIA/3/ALTAR84/CMH82A.1062//RISSA’S’/3/ACONCHI89	A-31906-0P-0P-0P-1A-7C-0C	INIA
24	QUC 3559-2009	SOOTY9/RASCON37//STORLOM	CGSS02Y00006S-2F1-12Y-0B-3Y-0B-2Y-0B	INIA
25	QUC 3501-2009	CMH85.797//CADO/BOOMER33/4/ARMENT//SRN3/NIGRIS4/3/CANELO9.1	CDSS02B01468T-0TOPB-0Y-0M-5Y-4M-04Y-0B	INIA
26	QUC 3449-2009	STORLOM/3/RASCON37/TARRO2//RASCON37/4/KIRKI1/HIMAN9/5/GLAS5/LOTUS4//SOMBRA20	CDSS02Y01082T-0TOPB-0Y-0M-11Y-0Y	INIA
27	QUC 3546-2009	CBC 509 CHILE/5/2*AJAIA16//HORA/JRO/3/GAN/4/ZAR	CDSS02Y01222T-0TOPB-0Y-0M-1Y-0Y	INIA
28	QUC 3425-2009	NACH’S’/CHEN’S’//RUFO’S’/ALD’S’/3/SQLA’S’/4/CD 64399-4P-2P-P//STN’S’/GOTE’S’	A-32116-0P-0P-1A-1C-1C-0C	INIA
29	QUC 3487-2009	TARRO1/TISOMA2//TARRO1/3/COMBDUCK2/4/SHAG9/BUTO17/6/CHEN1/TEZ/3/GUIL//CIT71/CII/4/SORA/PLATA12/5/STOT//ALTAR 84/ALD	CDSS02B00451S-0Y-0M-4Y-2M-04Y-0B	INIA
30	QUC 3504-2009	MINIMUS/COMB DUCK2//CHAM3/3/CANELO9/9/USDA595/3/D67.3/RABI//CRA/4/ALO/5/HUI/YAV1/6/ARDENTE/7/HUI/YAV79/8/POD9	CDSS02Y00259S-0Y-0M-24Y-0Y	INIA
31	QUC 3521-2009	TRN//21563/AA/3/BD2080/4/BD2339/5/RASCON37/TARRO2//RASCON37/6/AUK/GUIL//GREEN	XXX	INIA
32	QUC 3541-2009	HUBEI//SOOTY9/RASCON37/3/2*SOOTY9/RASCON37/4/CRAKE10/RISSA	CDSS02Y00587S-0Y-0M-16Y-0Y	INIA
33	QUC 3507-2009	ODIN15/WITNEK1//ISLOM1/5/TARRO/TISOMA2//TARRO1/3/COMB DUCK2/ALAS//4*COMB DUCK2/4/SHAG9/BUTO17	CDSS04Y00014S-21Y-0M-06Y-2M-1Y-0B	INIA

34	QUC 3448-2009	LLARETA INIA/3/ALTAR84/CMH82A.1062//RISSA'S'	A-32133-0P-0P-1A-1C-6C-0C	INIA
35	QUC 3462-2009	CAMAYO/GUANAY	CDSS01B00082S-19M-0M-0Y-0Y	INIA
36	QUC 3484-2009	GREEN38/BUSHEN4//PORTO3/4/ARMENT//SRN3/NIGRIS4/3/CANELO 9.1	CDSS02B00267S-0Y-0M-4Y-3M-04Y-0B	INIA
37	QUC 3544-2009	BCRIS/BICUM//LLARETA INIA/3/DUKEM_12/2*RASCON_21	CDSS99B01189T-0TOPY-0M-0Y-81Y-0M-0Y-1M-0Y	INIA
38	QUC 3453-2009	D94528/3/2*STOT//ALTAR 84/ALD/5/TARRO1/TISOMA2//TARRO1/3/COMB DUCK2/ALAS//4*COMB DUCK2/4/SHAG9/BUTO7	CDSS02B00639S-0Y-0M-5Y-4M-04Y-0B	INIA
39	QUC 3465-2009	CANELO8//SORA/2*PLATA12/4/STORLOM/3/RASCON37/TARRO2//RASCON37/5/RASCON39/TILO1	CDSS02Y01103T-0TOPB-0Y-0M-14Y-0Y	INIA
40	QUC 3491-2009	GUANAY/4/YAZI_1/AKAKI4//SOMAT3/3/AUK/GUIL//GREEN/5/NUS/SULA//5*NUS/4/SULA/RBCE_2/3/HUI//CIT71/CII	CDSS02B01013T-0TOPB-0Y-0M-3Y-1M-04Y-0B	INIA
41	QUC 3485-2009	ARAM7//CREX/ALLA/5/ENTE/MEX2//HUI/4/YAV1/3/LD357E/2*TC60//JO69/6/CANELO9.1/7/STORLOM/3/RASCON37/TARRO2//RASCON37	CDSS02B00340S-0Y-0M-23Y-1M-04Y-0B	INIA
42	QUC 3419-2009	VR.90D015/LLOYD/4/NACH'S'/CHEN'S'//RUFO'S'/3/SQLA'S'	A-32098-0P-0P-1A-1C-7C-0C	INIA
43	QUC 3403-2009	ALTAR84/CMH82A.1062//RISSA'S'/7/YEL'S'/BAR'S'/3/GR'S'/AFN//CR'S'/5/DOM'S'//CR'S'*2/3/SCO'S'/4/HORA/3/LAP76/GUIL'S'	A-32085-0P-0P-1A-1C-3C-0C	INIA
44	QUC 3538-2009	TOSKA 26/RASCON37//SNITAN/4/ARMENT//SRN3/NIGRIS4/3/CANELO 9.1	CDSS02Y00395S-0Y-0M-6Y-0Y	INIA
45	QUC 3456-2009	PLATA3//CREX/ALLA/3/YAZI10/4/STOT//ALTAR/COMB DUCK2//CHAM3/4/ALD/7/CHEN11/POC/TANTLO/5/ENTE/MEXI2//HUI/4/YAV1/3/LD357E/2*TC60//JO69/6/MINIMUS	CDSS02B00916T-0TOPB-0Y-0M-2Y-1M-04Y-0B	INIA
46	QUC 3503-2009	ODIN15/WITNEK1//ISLOM1	CDSS95Y00943T-A-1Y-0M-0Y-0B-2Y-0B-0BLR-3Y-0B	INIA
47	QUC 3506-2009	NUS/SULA//5*NUS/4/SULA/RBCE2/3/HUI//CIT71/CII/5/1A.1D 5+10-6/2*WB881//1A.1D 5+10-6/3*MOJO/3/BISU1/PATKA3	CDSS02B00286S-0Y-0M-8Y-1M-04Y-0B	INIA
48	QUC 3405-2009	ALTAR84/CMH82A.1062//RISSA'S'/3/ALTAR84/CMH82A.1062//RISSA'S'	A-32086-0P-0P-1A-1C-2C-0C	INIA

49	QUC 3407-2009	ALTAR84/CMH82A.1062//RISSA’S’/3CD64399-4P-2P-3P//STN’S’/GOTE’S’	A32090-0P-0P-1A-1C-6C-0C	INIA
50	Without Code	CRAME'S'/PLAC1485	A 24819-16P-5P-1P	INIA
51	Without Code	NACH'S'/CHEN'S'//RUFO'S'/ALD'S'/3/SQLA'S'	CD79634-1Y-040M-030YRL-2M-2YRL-0PAP	INIA
52	Without Code	ALTAR84/CMH82A1062//RISSA'S'/3/F3LOCAL	A 27743-0P-2P-1P-2P	INIA
53	Without Code	CD64399-4P-2P-3P´//STN'S'/GOTE'S'	A27191-0P-7P-2P-1P	INIA
54	Without Code	CD64399-4P-2P-3P/3/BGTO//SHWA'S'/MAL'S'	A27182-0P-3P-1P-1P	INIA
55	Without Code	POHO 1	CD90226-501M-503YRC-502B-0Y	INIA
56	Without Code	KUCUK	CD91B2620G-2M-030Y-030M	INIA
57	Without Code	PLAC3089//CHEN'S'/ALTAR	A29584-0P-1P-0P-0P-10P	INIA
58	Without Code	NACH'S'/CHEN'S'//RUFO'S'/ALD'S'/3/SULA'S'/4/F2M.DUR.P.1981-C6	A30226-0P-0P-0P-2P	INIA
59	Without Code	SORA/2*PLATA12//SRN3/NIGRIS4	CDSS96Y00460S-4Y-0M-0Y-1B-0Y-0B-0B-0BLR-2Y-0B	INIA
60	Without Code	SOMAT3.1//WODUCK/CHAM3/5/AJAIA16//HORA/JRO/3/GAN/4/ZAR	CDSS00Y01093T-0TOPB-2Y-0BLR-3Y-0B-0Y-0B	INIA
61	Without Code	AJAIA16//HORA/JRO/3/GAN/4/ZAR/5/SUOK7/6/STOT//ALTAR84/ALD	CDSS99B00778S-0TOPY-0M-0Y-129Y-0M-0Y-1B	INIA
62	Without Code	ALTAR84/STINT//SILVER45/3/GUANAY/4/GREEN 14//YAV10/AUK	CDSS99B01260T-0TOPY-0M-0Y-24Y-0M-0Y-0B	INIA
63	Without Code	SOMAT3/3/STOT//ALTAR84/ALD/4/FOCHA1/MUSK4	CDSS00Y01056T-0TOPB-17Y-0BLR-8Y-0B-0Y-1B	INIA
64	Without Code	STOT//ALTAR84/ALD/3/THB/CEP7780//2*MUSK4/4AUK/GUIL//GREEN	XXX	INIA
65	Without Code	DA-6 Black awns/3/Bcr//Memo/Goo	ICD96-0058-C-0AP-2AP-0AP-9AP-AP-2AP-0AP-1AP-AP	INIA
66	Without Code	1A.1D 5+10-5/3*MOJO/3/2*TARRO 2/MOJO 2//RASCON 33	CDSS96B01149M-0TOPY-4M-0Y-1B-0Y-0B-0B-1Y-0M-0Y	INIA
67	Without Code	SOOTY 9/RASCON 37/3/SOOTY 9/TARRO 1//AJAIA 2	CDSS97Y00565S-8Y-0M-0Y-0B-0B-1Y-0M	INIA
68	Without Code	ALTAR 84/STINT//SILVER 45/3/CAMAYO/5/CHEN 11/POC//TANTLO/4/ENTE/MEXI 2//HUI/3/YAV 1/GEDIZ	CDSS99B01112T-0TOPY-0M-0Y-4Y-0M-0Y-0B	INIA

69	Without Code	STOT//ALTAR84/ALD*2/3/AUK/GUIL//GREEN	CDSS00Y00786T-0TOPB-18Y-0BLR-6Y-0B-0Y-0B	INIA
70	Without Code	SOOTY9/RASCON37//SOMAT 3.1	CGSS02Y00003S-1F1-34Y-0B-5Y-0B	INIA
71	Without Code	SOOTY9/RASCON37//STOT//ALTAR//ALTAR84/ALD	CGSS02Y00002S-1F1-8Y-50Y-0B	INIA
72	QUC 3205-2006	GUAYACAN INIA/7/YEL'S'/BAR'S'/3/GR'S'/AFN//CR'S'/5/DOM'S'//CR'S'*2/GS'S'/3/	A 31322-0P-0P-0P-0P-1P	INIA
73	QUC 3311-2006	ALTAR84/CMH82A.1062//RISSA'S'/3/GUAYACAN INIA	A-31096-0P-0P-0P-0P-1P-0C	INIA
74	QUC 3439-2006	TEZ'S'/YAV79//HUI'S'/3/SOMO'S'/7/21563/3/LKE/LD399//21563/AA'S'//FG'S'/6/EIP/3/GS'S'/TC60//MEXI'S'/4/SHWA'S'/REN/8/ALTAR	A-31576-0P-0P-0P-0P-1A-0C	INIA
75	QUC 3469-2007	CRANE'S'/PLAC1485/3/ALTAR84/STINT'S'//SILVER	A-31590-0P-0P-0P-0P-1A-0C	INIA
76	QUC 3488-2007	NACH'S'/CHEN'S'//RUFO'S'/ALD'S'/3/SQLA'S'/4/NACH'S'/CHEN'S'//RUFO'S'/ALD'S'/3/SQLA'S'	A-31603-0P-0P-0P-0P-2A-0C	INIA
77	QUC 3509-2007	ATES 2-D/7/YEL'S'/BAR'S'/3/GR'S'/AFN//CR'S'/5/DOM'S'//CR'S'*2/GS'S	A-31617-0P-0P-0P-0P-1A-0C	INIA
78	QUC 3324-2007	ALTAR84/STINT'S'//SILVER/3/ETH-LRBR A-133/3*ALTAR84	A-31634-0P-0P-0P-0P-1A-0C	INIA
79	QUC 3538-2007	YEL'S'/BAR'S'/3/GR'S'/AFN//CR'S'/5/DOM'S'//CR'S'*2/GS'S'/3/SCO'S'/4/HORA	A-31659-0P-0P-0P-0P-1A-0C	INIA
80	QUC 3547-2007	YEL'S'/BAR'S'/3/GR'S'/AFN//CR'S'/5/DOM'S'//CR'S'*2/GS'S'/3/SCO'S'/4/HORA	A-31660-0P-0P-0P-0P-2A-0C	INIA
81	QUC 3412-2007	YEL'S'/BAR'S'/3/GR'S'/AFN//CR'S'/5/DOM'S'//CR'S'*2/GS'S'/3/SCO'S'/4/HORA	A-31662-0P-0P-0P-0P-1A-0C	INIA
82	QUC 3555-2007	NACH'S'/CHEN'S'//RUFO'S'/ALD'S'/3/SQLA'S'/4/CRANE'S'/PLAC1485	A-31677-0P-0P-0P-0P-1A-0C	INIA
83	QUC 3556-2007	ALTAR84/CMH82A.1062//RISSA'S'/4/QFN'S'/KILL'S'	A-31690-0P-0P-0P-0P-3A-0C	INIA
84	QUC 3584-2007	POHO1/4/ALTAR84/CMH84/CMH82A.1062//RISSA'S'/3/ACONCHI89	A-31732-0P-0P-0P-0P-1A-0C	INIA
85	QUC 3597-2007	ALTAR84/STINT'S'//SILVER/7/YEL'S'/BAR'S'/3/GR'S'/AFN//CR'S'/5/DOM'S'//CR'S'*2/GS'S'/3/SCO'S'/4/HORA/6/LAP76/GUIL'S'	A-31752-0P-0P-0P-0P-3A-0C	INIA
86	QUC 3661-2007	CANELO 9.1//SOOTY 9/RASCON 37	CDSS97Y00285S-1Y-0M-0Y-0B-0B-1Y-0M	INIA
87	QUC 3677-2007	E90035/2*YUAN 1/4/BISU1//CHEN/TEZ/3/HUI//CIT71/CII/5/STOT//ALTAR84/ALD	CDSS99B00866S-0TOPY-0M-0Y-49Y-0M-0Y-1B	INIA

88	QUC 3327-2010	STOT//ALTAR 84/ALD	CD91Y636-1Y-040M-030Y-1M-0Y-0B-1Y-0B	INIA
89	QUC 3332-2010	SOMAT_3/PHAX_1//TILO_1/LOTUS_4/3/SOOTY_9/RASCON_37	CDSS01B00473S-0Y-0M-13Y-0Y	INIA
90	QUC 3333-2010	HYDRANASSA30/SILVER_5/3/AUK/GUIL//GREEN/10/PLATA_10/6/MQUE/4/USDA573//QFN/AA_7/3/ALBA-D/5/AVO/HUI/7/PLATA_13/8/THKNEE_11/9/CHEN/ALTAR 84/3/HUI/POC//BUB/RUFO/4/FNFOOT	CDSS02Y00152S-0Y-0M-16Y-0Y	INIA
91	QUC 3334-2010	PLATA_6/GREEN_17//SNITAN/4/YAZI_1/AKAKI_4//SOMAT_3/3/AUK/GUIL//GREEN	CDSS02Y00369S-0Y-0M-31Y-0Y	INIA
92	QUC 3335-2010	RASCON_37/2*TARRO_2/3/AJAIA_12/F3LOCAL(SEL.ETHIO.135.85)//PLATA_13/4/SORA/2*PLATA_12//SOMAT_3	CDSS02Y00378S-0Y-0M-7Y-0Y	INIA
93	QUC 3336-2010	RASCON_38/SNITAN/9/USDA595/3/D67.3/RABI//CRA/4/ALO/5/HUI/YAV_1/6/ARDENTE/7/HUI/YAV79/8/POD_9	CDSS02Y00379S-0Y-0M-13Y-0Y	INIA
94	QUC 3337-2010	PORRON_1/MRB589_5//PORRON_1/3/SORA/2*PLATA_12//SOMAT_3/4/RASCON_37/2*TARRO_2	CDSS02Y01037T-0TOPB-0Y-0M-3Y-0Y	INIA
95	QUC 3338-2010	LHNKE/HCN//PATA_2/3/SOMAT_4/INTER_8/5/CREX//BOY/YAV_1/3/PLATA_6/4/PORRON_11	CDSS02Y01190T-0TOPB-0Y-0M-5Y-0Y	INIA
96	QUC 3339-2010	SOMAT_3/PHAX_1//TILO_1/LOTUS_4/3/2*STOT//ALTAR 84/ALD	CDSS02Y01267T-0TOPB-0Y-0M-8Y-0Y	INIA
97	QUC 3341-2010	SORA/2*PLATA_12//SOMAT_3/3/STORLOM/4/BICHENA/AKAKI_7	CDSS02Y01279T-0TOPB-0Y-0M-29Y-0Y	INIA
98	QUC 3343-2010	STORLOM/3/SOMAT_3/PHAX_1//TILO_1/LOTUS_4	CDSS02B00004S-0Y-0M-24Y-1M-04Y-0B	INIA
99	QUC 3344-2010	CHEN_1/TEZ/3/GUIL//CIT71/CII/4/SORA/PLATA_12/5/STOT//ALTAR 84/ALD/6/SOMAT_3/PHAX_1//TILO_1/LOTUS_4	CDSS02B00021S-0Y-0M-25Y-2M-04Y-0B	INIA
100	QUC 3345-2010	VRKS_3/7/ENTE/MEXI_2//HUI/4/YAV_1/3/LD357E/2*TC60//JO69/5/BISU/6/RYPS26_2/10/PLATA_10/6/MQUE/4/USDA573//QFN/AA_7/3/ALBA-D/5/AVO/HUI/7/PLATA_13/8/THKNEE_11/9/CHEN/ALTAR 84/3/HUI/POC//BUB/RUFO/4/FNFOOT	CDSS02B00145S-0Y-0M-9Y-1M-04Y-0B	INIA

101	QUC 3346-2010	SOOTY_9/RASCON_37/3/SOOTY_9/TARRO_1//AJAIA_2/10/PLATA_10/6/MQUE/4/USDA573//QFN/AA_7/3/ALBA-D/5/AVO/HUI/7/PLATA_13/8/THKNEE_11/9/CHEN/ALTAR 84/3/HUI/POC//BUB/RUFO/4/FNFOOT	CDSS02B00379S-0Y-0M-24Y-4M-04Y-0B	INIA
102	QUC 3347-2010	SOOTY_9/RASCON_37//TILO_1/LOTUS_4/9/USDA595/3/D67.3/RABI//CRA/4/ALO/5/HUI/YAV_1/6/ARDENTE/7/HUI/YAV79/8/POD_9	CDSS02B00380S-0Y-0M-4Y-3M-04Y-0B	INIA
103	QUC 3348-2010	SHAG_21/DIPPER_2//PATA_2/6/ARAM_7//CREX/ALLA/5/ENTE/MEXI_2//HUI/4/YAV_1/3/LD357E/2*TC60//JO69/7/ARMENT//SRN_3/NIGRIS_4/3/CANELO_9.1	CDSS02B00443S-0Y-0M-15Y-3M-04Y-0B	INIA
104	QUC 3349-2010	TADIZ/9/USDA595/3/D67.3/RABI//CRA/4/ALO/5/HUI/YAV_1/6/ARDENTE/7/HUI/YAV79/8/POD_9	CDSS02B00455S-0Y-0M-6Y-4M-04Y-0B	INIA
105	QUC 3351-2010	LOTUS_5/SORD_1/3/CANELO_8//SORA/2*PLATA_12/9/USDA595/3/D67.3/RABI//CRA/4/ALO/5/HUI/YAV_1/6/ARDENTE/7/HUI/YAV79/8/POD_9	CDSS02B00770T-0TOPB-0Y-0M-17Y-2M-04Y-0B	INIA
106	QUC 3352-2010	SOMAT_4/SILVER_1/4/STORLOM/3/RASCON_37/TARRO_2//RASCON_37/5/PATKA_4/PLATA_16	CDSS02B00964T-0TOPB-0Y-0M-7Y-1M-04Y-0B	INIA
107	QUC 3353-2010	SNITAN/5/AJAIA_12/F3LOCAL(SEL.ETHIO.135.85)//PLATA_13/3/SOMAT_3/4/SOOTY_9/RASCON_37/6/SNITAN	CDSS02B01019T-0TOPB-0Y-0M-3Y-4M-04Y-0B	INIA
108	QUC 3354-2010	DIPPER_2/BUSHEN_3//SNITAN/3/SOMAT_3/PHAX_1//TILO_1/LOTUS_4/5/PATIN_7//HUI/YAV79/3/AJAIA_12/F3LOCAL(SEL.ETHIO.135.85)//PLATA_13/4/RASCON_37/GREEN_2	CDSS02B01086T-0TOPB-0Y-0M-2Y-2M-04Y-0B	INIA
109	QUC 3355-2010	MINIMUS/COMB DUCK_2//CHAM_3/3/RCOL*2/4/SOMAT_4/INTER_8	CDSS02B01108T-0TOPB-0Y-0M-5Y-4M-04Y-0B	INIA
110	QUC 3356-2010	TARRO_1/2*YUAN_1//AJAIA_13/YAZI/3/SOMAT_3/PHAX_1//TILO_1/LOTUS_4/4/CANELO_8//SORA/2*PLATA_12	CDSS02B01143T-0TOPB-0Y-0M-3Y-4M-04Y-0B	INIA
111	QUC 3357-2010	CF4-JS 40//SOOTY_9/RASCON_37/4/CNDO/PRIMADUR//HAI-OU_17/3/SNITAN	CDSS02B01336T-0TOPB-0Y-0M-1Y-3M-04Y-0B	INIA

112	QUC 3358-2010	CF4-JS 40/10/PLATA_10/6/MQUE/4/USDA573//QFN/AA_7/3/ALBA-D/5/AVO/HUI/7/PLATA_13/8/THKNEE_11/9/CHEN/ALTAR 84/3/HUI/POC//BUB/RUFO/4/FNFOOT/11/PATIN_7//HUI/YAV79/3/AJAIA_12/F3LOCAL(SEL.ETHIO.135.85)//PLATA_13/4/RASCON_37/GREEN_2	CDSS02B01339T-0TOPB-0Y-0M-4Y-1M-04Y-0B	INIA
113	QUC 3359-2010	HUBEI//SOOTY_9/RASCON_37/3/2*SOOTY_9/RASCON_37/4/SOOTY_9/RASCON_37/5/SOOTY_9/RASCON_37	CDSS02B01428T-0TOPB-0Y-0M-1Y-3M-04Y-0B	INIA
114	QUC 3361-2010	CMH79.1159/POC//CRAKE_10/RISSA/9/USDA595/3/D67.3/RABI//CRA/4/ALO/5/HUI/YAV_1/6/ARDENTE/7/HUI/YAV79/8/POD_9	CDSS02B01456T-0TOPB-0Y-0M-9Y-4M-04Y-0B	INIA
115	Without Code	PATKA_7/YAZI_1//FICHE_6/3/SOOTY_9/RASCON_37/4/SOMAT_3/PHAX_1//TILO_1/LOTUS_4/5/SOOTY_9/RASCON_37	CDSS02B01161T-0TOPB-0Y-0M-5Y-1M-04Y-0B	INIA
116	Without Code	WDRAIL_1/TOSKA_26//PLATA_6/GREEN_17/3/SORA/2*PLATA_12//SOMAT_3/4/SORA/2*PLATA_12//RASCON_37	CDSS02B01194T-0TOPB-0Y-0M-1Y-1M-04Y-0B	INIA
117	Without Code	RASCON_37/4/MAGH72/RUFO//ALG86/RU/3/PLATA_16/5/PORTO_3*2/6/ARMENT//SRN_3/NIGRIS_4/3/CANELO_9.1	CDSS02B01204T-0TOPB-0Y-0M-8Y-3M-04Y-0B	INIA
118	Without Code	D86135/ACO89//PORRON_4/3/SNITAN/4/ALTAR 84 ALTO_1/RASCON_19/5/D86135/ACO89//PORRON_4/3/SNITAN	CDSS02B01255T-0TOPB-0Y-0M-3Y-2M-04Y-0B	INIA
119	Without Code	PON_3//SORA/2*PLATA_12/3/SNITAN/SOMAT_3//FULVOUS_1/MFOWL_13/4/BICHENA/AKAKI_7	CDSS02B01273T-0TOPB-0Y-0M-4Y-1M-04Y-0B	INIA
120	Without Code	P91.272.3.1/2*MEXI75//2*SOOTY_9/RASCON_37	CDSS02B01532T-0TOPB-0Y-0M-1Y-1M-04Y-0B	INIA
121	Without Code	RISSA/GAN//POHO_1/3/PLATA_3//CREX/ALLA*2/4/ARMENT//SRN_3/NIGRIS_4/3/CANELO_9.1	CDSS02B01131T-0TOPB-0Y-0M-5Y-4M-04Y-0B	INIA
122	Without Code	TARRO_1/2*YUAN_1//AJAIA_13/YAZI*2/9/USDA595/3/D67.3/RABI//CRA/4/ALO/5/HUI/YAV_1/6/ARDENTE/7/HUI/YAV79/8/POD_9	CDSS02B01141T-0TOPB-0Y-0M-3Y-4M-04Y-0B	INIA
123	Without Code	TARRO_1/2*YUAN_1//AJAIA_13/YAZI*2/4/ARMENT//SRN_3/NIGRIS_4/3/CANELO_9.1	CDSS02B01142T-0TOPB-0Y-0M-1Y-3M-04Y-0B	INIA

124	Without Code	MINIMUS/COMB DUCK_2//CHAM_3/3/FICHE_6/4/MOJO/AIRON/5/SOMAT_3.1/6/CHEN/ALTAR 84/3/HUI/POC//BUB/RUFO/4/FNFOOT/5/TILO_1/LOTUS_4	CDSS02B01159T-0TOPB-0Y-0M-3Y-2M-04Y-0B	INIA
125	Without Code	GARAVITO_2/4/MAGH72/RUFO//ALG86/RU/3/PLATA_16/5/PATA_2/6/SOMAT_4/INTER_8/7/SRN_3/NIGRIS_4//SHIP_1	CDSS02B01199T-0TOPB-0Y-0M-1Y-2M-04Y-0B	INIA
126	Without Code	SORA/2*PLATA_12//RASCON_37/4/ARMENT//SRN_3/NIGRIS_4/3/CANELO_9.1/5/KITTI_1/DUKEM_4//5*KITTI_1/3/ADAMAR_15	CDSS02B01236T-0TOPB-0Y-0M-4Y-1M-04Y-0B	INIA
127	Without Code	LHNKE/HCN//PATA_2/3/SOMAT_4/INTER_8/4/PLATA_6/GREEN_17//SNITAN	CDSS02B01243T-0TOPB-0Y-0M-5Y-1M-04Y-0B	INIA
128	Without Code	D86135/ACO89//PORRON_4/3/SNITAN/4/TATLER_1/TARRO_1//HYDRANASSA30/SILVER_5/5/D86135/ACO89//PORRON_4/3/SNITAN	CDSS02B01253T-0TOPB-0Y-0M-6Y-4M-04Y-0B	INIA
129	Without Code	PLATA_6/GREEN_17/3/CHEN/AUK//BISU*2/5/PLATA_3//CREX/ALLA/3/SOMBRA_20/4/SILVER_14/MOEWE	CDSS02B01266T-0TOPB-0Y-0M-10Y-3M-04Y-0B	INIA
130	Without Code	POD_20//SULA/ACO89/3/SORA/2*PLATA_12//SOMAT_3/4/PATKA_4/THKNEE_9//CABECA_1	CDSS02B01271T-0TOPB-0Y-0M-7Y-3M-04Y-0B	INIA
131	Without Code	RASCON_37/2*TARRO_2/3/AJAIA_12/F3LOCAL(SEL.ETHIO.135.85)//PLATA_13/4/SORA/2*PLATA_12//SOMAT_3/5/EUPODA_3/SLA_2//MINIMUS	CDSS02B01274T-0TOPB-0Y-0M-3Y-2M-04Y-0B	INIA
132	Without Code	THKNEE_11/SNITAN*2//SOMAT_4/INTER_8	CDSS02B01283T-0TOPB-0Y-0M-2Y-3M-04Y-0B	INIA
133	Without Code	TOPDY_18/FOCHA_1//ALTAR 84/3/AJAIA_12/F3LOCAL(SEL.ETHIO.135.85)//PLATA_13/4/SOMAT_3/GREEN_22/5/VRKS_3/3/AJAIA_12/F3LOCAL(SEL.ETHIO.135.85)//PLATA_13	CDSS02B01285T-0TOPB-0Y-0M-5Y-1M-04Y-0B	INIA
134	Without Code	TOSKA_26/RASCON_37//SNITAN*2/4/ARMENT//SRN_3/NIGRIS_4/3/CANELO_9.1	CDSS02B01286T-0TOPB-0Y-0M-2Y-4M-04Y-0B	INIA
135	Without Code	1A.1D 5+10-6/3*MOJO//RCOL/3/SNITAN/SOMAT_3//FULVOUS_1/MFOWL_13/4/INTER_16/SNITAN	CDSS02B01292T-0TOPB-0Y-0M-1Y-2M-04Y-0B	INIA
136	Without Code	CMH85.797//DUKEM_12/2*RASCON_21/9/USDA595/3/D67.3/RABI//CRA/4/ALO/5/HUI/YAV_1/6/ARDENTE/7/HUI/YAV79/8/POD_9	CDSS02B01465T-0TOPB-0Y-0M-16Y-4M-04Y-0B	INIA

137	Without Code	WELLO 1/DZ 1052/3/TARRO_1/TISOMA_2//TARRO_1/4/2*DUKEM_12/2*RASCON_21	CDSS02B01524T-0TOPB-0Y-0M-1Y-2M-04Y-0B	INIA
138	Without Code	ADAMAR/4/CHEN_1/TEZ/3/GUIL//CIT71/CII/5/SORA/2*PLATA_12//SOMAT_3/6/MINIMUS/COMB DUCK_2//CHAM_3/3/GREEN_19	CDSS02B01246T-0TOPB-0Y-0M-9Y-4M-04Y-0B	INIA
139	Without Code	Mrb5	XXX	ICARDA
140	Without Code	Azeghar-1	ICD92-0511-MABL-0AP-16AP-0TR-9AP-0AP	ICARDA
141	Without Code	Gcn/4/D68-1-93A-1A//Ruff/Fg/3/Mtl-5	ICD95-1302-C-3AP-0AP-1AP-0AP-5AP-AP-3AP-0AP	ICARDA
142	Without Code	Gdr2	xxx	ICARDA
143	Without Code	Bcr/Gro1//Mgnl1	ICD97-0396-T-1AP-AP-6AP-0AP-6AP-AP	ICARDA
144	Without Code	Krf	XXX	ICARDA
145	Without Code	Mgnl3/Aghrass2	ICD99-0015-C-8AP-AP-6AP-AP	ICARDA
146	Without Code	Bicrederaa1/3/Stj3//Dra2/Bcr	ICD99-0861-C-14AP-AP-14AP-AP	ICARDA
147	Without Code	Bcr/Lks4/Mrf1/Stj2	ICD99-0886-C-17AP-AP-3AP-AP	ICARDA
148	Without Code	Mrf/Stj2//Bcrch1	ICD99-0027-C-0AP-14AP-AP-7AP-AP	ICARDA
149	Without Code	Stj3//Dra2/Bcr/3/Ter-3	ICD99-0036-C-0AP-21AP-AP-13AP-AP	ICARDA
150	Without Code	Hau	XXX	ICARDA
151	Without Code	Waha	XXX	ICARDA
152	Without Code	Kulrengi/Balikcil8//Aghrass2	ICD99-0211-T-2AP-AP-11AP-AP	ICARDA
153	Without Code	Ter-1/3/Mrf1//Mrb16/Ru	ICD99-1024-T-0AP-6AP-AP-6AP-AP	ICARDA
154	Without Code	Ter-1/3/Stj3//Bcr/Lks4	ICD99-1036-T-0AP-1AP-AP-9AP-AP	ICARDA
155	Without Code	Azegar-2//Ch1/F1 13	ICD98-0493-W-AP-2AP-0AP-6AP-AP-6AP-AP	ICARDA
156	Without Code	Icasyr-2	ICD95-0169-C-0AP-2AP-0AP-4AP-0AP	ICARDA
157	Without Code	Ammar-1	ICD94-0918-C-12AP-0AP-4AP-0AP-4AP-0AP	ICARDA
158	Without Code	Mrf1/Stj2//Gdr2/Mgnl1	ICD01-0946-C-AP-13AP-TR	ICARDA
159	Without Code	Aghrass-1/3/Mrf1//Mrb16/Ru	ICD00-0834-C-32AP-AP-6AP-TR	ICARDA

160	Without Code	Gsbl1/4/D68-1-93A-1A//Ruff/Fg/3/Mtl5/5/Wdz6/Gil4	ICD01-0995-T-AP-2AP-AP	ICARDA
161	Without Code	Bicrederaa-1/Azeghar-2	ICD01-1060-T-AP-1AP-TR	ICARDA
162	Without Code	13376/Bcrch1//Ossl1/Stj5	ICD00-0360-T-8AP-AP-2AP-TR	ICARDA
163	Without Code	Ysf-1/3/Altar84/Stn//Wdz-2	ICD00-0999-T-7AP-AP-2AP-AP	ICARDA
165	Without Code	Azn-1//Ossl-1/Gdfl	ICD00-0898-H-18AP-AP-6AP-TR	ICARDA
166	Without Code	Azn-1/3/Mrf2//Bcr/Gro1	ICD00-0900-H-23AP-AP-2AP-TR	ICARDA
167	Without Code	Bcrch-1/3/Mrf2//Bcr/Gro1	ICD00-0928-H-4AP-AP-4AP-TR	ICARDA
168	Without Code	Msbl-1//Krf/Hcn	ICD95-1133-T-0AP-1AP-0AP-3AP-0TR-2AP-AP	ICARDA
169	Without Code	Adnan-2	ICD99-0846-C-13AP-0AP-5AP-AP-6AP-AP	ICARDA
170	Without Code	Bcrch1//Mrf1/Stj2	ICD97-0494-T-13AP-AP-5AP-0AP-16AP-AP	ICARDA
171	Without Code	Ter-1//Mrf1/Stj2	ICD99-0866-C-0AP-5AP-AP-5AP-AP	ICARDA
172	Without Code	Mgnl3/Ainzen-1	ICD98-0043-C-3AP-0AP-8AP-AP-4AP-AP	ICARDA
173	Without Code	Sjt3//Bcr/Lks4/3/Ter-3	ICD99-0091-T-3AP-AP-6AP-AP	ICARDA
174	Without Code	HFN94N-37/Mrb5/3/Brch/T.dic 20017//Hcn	ICD95-0638-T-0AP-3AP-0AP-4AP-0TR-3AP-AP-2AP-0AP-2AP-AP	ICARDA
175	Without Code	Bcr/Lks4/4/BEZAIZ-SHF//SD-19539/Waha/3/Stj/Mrb3/5/Ossl1/Stj5	ICD00-0389-T-AP-5AP-AP	ICARDA
176	Without Code	Cl115/Azn1/5/Villemur/3/Lahn//Gs/Stk/4/Dra2/Bcr	ICD00-0432-T-AP-13AP-AP	ICARDA
177	Without Code	Aghrass-1/3/HFN94N-8/Mrb5//Zna-1	ICD00-1085-T-10AP-AP-10AP-AP	ICARDA
178	Without Code	Ter-2/3/HFN94N-8/Mrb5//Zna-1	ICD00-1090-T-2AP-AP-2AP-AP-TR	ICARDA
179	Without Code	Villemur/3/Lahn//Gs/Stk/4/Dra2/Bcr/Lks4/4/BEZAIZ-SHF//SD-19539/Waha/3/Sjt/Mrb3	ICD00-0388-T-AP-12AP-AP-1AP-TR	ICARDA
180	Without Code	Aghrass-1/Bezaiz98-1	ICD00-0018-T-19AP-AP-2AP-TR	ICARDA
181	Without Code	Massara-1/4/Aus1/3/Scar/Gdo VZ579//Bit	ICD95-0916-C-2AP-0AP-3AP-0AP-12AP-AP-12AP-0AP	ICARDA
182	Without Code	Ouasbar-2	ICD96-0746-C-3AP-0AP-6AP-0AP	ICARDA
183	Without Code	Razzak/4/21563/AA//Fg/3/D68-10-2A-2A-1A/5/Aghrass2	ICD99-0368-T-11AP-AP-9AP-AP	ICARDA
184	Without Code	Akaki7/Lotus4//Himan9	XXX	ICARDA

185	Without Code	Altar84/Cas	XXX	ICARDA
186	Without Code	Tapdy/Rascon33	XXX	ICARDA
187	Without Code	ETH-LRBRA1-138(74)/3*Altar84	XXX	ICARDA
